# Significance of vasculopathy in IgA nephropathy patients with regard to Oxford classification and immunostaining findings: a single center experience

**DOI:** 10.12861/jrip.2013.16

**Published:** 2013-06-01

**Authors:** Hamid Nasri, Muhammed Mubarak

**Affiliations:** ^1^Department of Nephrology, Division of Nephropathology, Isfahan University of Medical Sciences, Isfahan, Iran; ^2^Department of Histopathology, Sindh Institute of Urology and Transplantation (SIUT), Karachi, Pakistan

**Keywords:** Immunoglobulin A nephropathy, Oxford classification, Vasculopathy

## Abstract

**Introduction:** immunoglobulin A nephropathy is the most common primary glomerulonephritis around the world. It remains poorly understood whether vascular pathology plays an important role in the progression of IgAN.

**Objectives:** Since very few studies regarding vascular disorders in IgAN are published, we aimed to evaluate the role of vascular disease in a group of IgAN patients, especially with regard to Oxford classification, immunostaining findings and various laboratory and clinical data.Patients and Methods: The study was conducted from July 2009 to March 2013. All kidney biopsies were prepared for light and immunofluorescence microscopy.

**Results:** Of the 136 patients, 94 (69%) were male. Of the 136 kidney biopsies, 37 patients had crescents, 2 had lesions of thrombotic microangiopathy (TMA), 10 had fibrinoid necrosis and one patient had morphologic lesions of small vessel vasculitis (with negative ANCA). In this study, there was no significant difference in scores of arteriolosclerosis and intimal fibrosis between males and females. There was a significant correlation between scores of arteriolosclerosis with serum creatinine. This correlation for scores of intimal fibrosis of interlobular artery was also significantly positive. The correlation of arteriolosclerosis with amount of proteinuria was significantly positive too.

**Conclusion:** The association of vasculopathy with serum creatinine and amount of proteinuria further support the role of vasculopathy in aggravation of IgAN. We also had different results with regard to the prevalence of TMA in previous published studies, which needs further investigation in larger series of IgAN patients.

**Implication for health policy/practice/research/medical education:**

The association of vasculopathy with serum creatinine and the level of proteinuria further support the role of vasculopathy in aggravation of IgAN. We also had different result in the prevalence of thrombotic microangiopathy with previous study, which needs further investigation in larger series of IgAN patients.

## Introduction

Immunoglobulin A nephropathy (IgAN) is the most common primary glomerulonephritis around the world (1). Patients with IgAN have variable clinical courses and during the past decade much attention has been focused toward factors having prognostic significance (1,2).Various morphologic lesions have been assessed for predicting progression of IgAN. However, after publication of Oxford classification of IgAN, four morphologic lesions, known as MEST variables, have been selected for the prediction of renal prognosis of IgAN: mesangial hypercellularity (M), endocapillary proliferation (E), segmental sclerosis or adhesion (S), and tubular atrophy/interstitial fibrosis (T) (3,4). Many investigations have focused on risk factors for kidney insufficiency in IgAN. Whether, other renal morphologic variables may have prognostic significance, is of much interest recently. Presence of extracapillary proliferation (1-3), location of deposited immunoglobulins (mesangial *versus*mesangio-capillary), have been suggested as having prognostic significance (1-3). However, it remains poorly understood whether vascular pathology plays an important role in the progression of IgAN. In a study to evaluate the role of vascular disease in the progression of IgAN by morphometric analysis in 71 IgAN patients, Katafuchi *et al*. observed that hypertension and vessel area were equally important as predictors of glomerular sclerosis and IgAN progression (4). A recent study in Japan, revealed that histological grade involving also vasculopathy at the initial biopsy was found to be a reliable parameter to predict IgAN progression (5,6).

## Objectives

Very few studies regarding vascular disorders in IgAN are published. We therefore aimed to assess the role of vascular disease in a group of IgAN patients, especially with regard to Oxford classification, immunostaining findings and various laboratory and clinical data.

## Patients and Methods

This study was conducted according to the Oxford classification of IgAN (1,4) from July 2009 to March 2013.

### Definition of IgAN

The pathologic diagnosis of IgAN necessitates the observation of IgA-dominant mesangial or mesangial-capillary immune deposits through immunofluorescence (IF) microscopy in the absence of C1q deposits (1,2). The immune deposits were semi-quantified from 0 to 3+ positive bright. The biopsy specimens were sent to a reference laboratory. None of the patients underwent treatment before the biopsy. Those biopsies that contained less than 8 glomeruli were excluded from this study. None of the patientshad a history of collagen vascular diseasesor liver cirrhosis.

### Histologic data

All kidney biopsies were prepared for light and direct IF microscopy. First, we performed an IF review, then we evaluated the glass slides. After an IF diagnosis of IgAN, we reviewed the histopathology glass slides to describe the four morphologic variables of Oxford classification and other morphologic lesions.

### Definitions of morphologic variables of IgAN and MEST (Oxford classification)

We estimated the presence of mesangial hypercellularity (M), endocapillary proliferation (E**)**, segmental glomerulosclerosis (S) and the proportion of tubular atrophy and interstitial fibrosis, IF/TA (T), as defined in the Oxford classification (1-3).

### Vascular definitions

**Figure 1:**
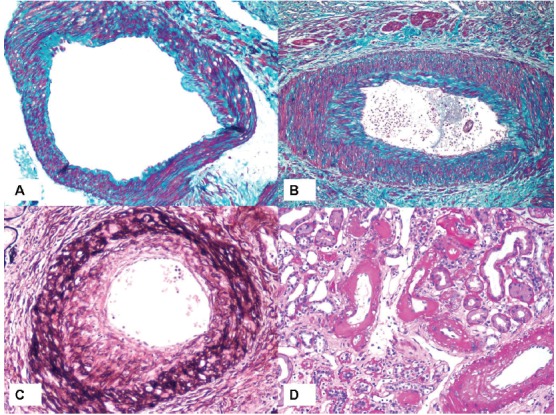
The criteria for scoring the vascular lesions of arteriolosclerosis and intimal thickening of arteries. A) High-power view of an arcuate artery showing no intimal thickening and widely patent lumen. The media of the vessel wall stains red on this staining. (Masson’s trichrome, ×400). B) This arcuate artery shows intimal thickening shown as a green layer facing the lumen. The thickness of the intima is less than the thickness of the media in this vessel, thus scoring as 1. (Masson’s trichrome, ×400). C) This artery also shows intimal thickening but the thickness of the intima is more than the thickness of the media in this vessel. The intimal score for this vessel is 2. (Jones methenamine silver stain, ×400). D) There is prominent arteriolosclerosis involving almost all arterioles in this field. This will be scored as score 3 in Oxford classification of IgA nephropathy. (Period acid-Schiff stain, ×400).

Intimal thickening was scored by comparing the thickness of the intima to that of the media in the samesegment of vessel. Intima was scored variously as normal (score: 0), andthickened to more or less than the thickness ofthe media (score: 2 or 1, respectively). Arteriolosclerosis (arteriolar hyaline) was noted as the proportion of arterioles affected and was scored according to the following categorization; normalarterioles (score: 0), 1-25% affected arterioles (score: 1), 26-50% affected arterioles (score: 2) and >50% affected arterioles (score: 3) (1-3). The scoring criteria are illustrated in Figure 1.

### Clinical and laboratory data

We reviewed the patients’ medical records to obtain various demographic, clinical and laboratory data at the time of their biopsies and for follow-up. We gathered the following data at the time of biopsy: race, gender, age, serum creatinine and proteinuria (based on a 24-hour urine collection).

### Ethical issues

(1) The research followed the tenets of the Declaration of Helsinki; (2) informed consent was obtained; and (3) the research was approved by the institutional review board.

### Statistical analysis

We determined the frequency, mean values and standard deviations of the categorical and continuous variables. The statistical significance of the differences between the male and female groups was done using the Mann-Whitney U test. We used the Spearman’s correlation coefficient to check the correlations. We used a computer program (SPSS version 16.0, Chicago, IL) for statistical analysis. P<0.05 was considered statistically significant.

## Results

### Population Characteristics

In our observational study, we enrolled a total of 136 IgAN patients’ biopsies.

### Prevalence

Of the 136 patients, 94 (69%) were male. The mean age of the patients was 37.6±13 years. The morphologic variables of the Oxford classification are illustrated in Table 1. The mean proteinuria was 1725±1247 mg/day (median=1500 mg/day). The mean number of glomeruli in all renal biopsies was 14.8±7.3. Table 2, shows the frequency and intensity scoring of antibody deposition. In all biopsies, the mean number of totally sclerotic glomeruli was 2.5±3.2 (median=2). Also, the mean of serum creatinine was 1.6±1.5 mg/dL (median=1.2 mg/dL). Of the 136 kidney biopsies, 37 (27.2%) patients had crescents, 2 (1.5%) had lesions of thrombotic microangiopathy (TMA), 10 (7.3%) had fibrinoid necrosis and one (0.7%) patient had morphologic lesions of small vessel vasculitis (with negative ANCA).

**Table 1:** Morphologic variables of Oxford classification.

Oxford-MEST variables N=136	Number	Percent
**M(0/1)**	46/90	33.8/66.2
**E(0/1)**	90/46	66.2/33.8
**S(0/1)**	48/88	35.3/64.7
**T(0/1/2)**	67/45/23	49.3/33.8/16.9

**Table 2:** Frequency of antibody and complement deposition intensity score.

Antibody deposition intensity N=136	0	+	++	+++
**IgA**	0	0	52	84
**IgG**	86	35	14	1
**IgM**	89	37	10	0
**C3**	33	50	37	16

Tables 3 and 4 show the frequency distribution of scores of arteriolosclerosis and intimal fibrosis of interlobular arteries.

**Table 3:** Frequency distribution of arteriolosclerosis scores.

Arteriolosclerosis Score	Number	Percent
**0**	71	52.2
**1**	38	27.9
**2**	27	19.9
**3**	0	0

**Table 4:** Frequency distribution of intimal thickening score of interlobular arteries.

Thickness of the intima Score	Number	Percent
**0**	79	58.1
**1**	56	41.2
**2**	1	0.7

### Gender differences

In this study, there was no significant difference in the scores of arteriolosclerosis and of intimal fibrosis between males and females (p>0.05).

### Correlations

There was significant correlation between scores of arteriolosclerosis with serum creatinine (r= 0.291, p= 0.001). This correlation for scores of intimal fibrosis of interlobular artery was also significantly positive (r= 0.339, p<0.001).

While, there was no significant association between proteinuria and scores of intimal fibrosis of interlobular arteries (P= N.S), the correlation of arteriolosclerosis with amount of proteinuria was significantly positive (r= 0.192, p<0.025).

Table 5  shows the association of morphologic variables of Oxford classification with scores of arteriolosclerosis and intimal fibrosis of interlobular arteries. Similarly, Table 6  shows the association of deposited antibodies intensity score with scores of arteriolosclerosis and intimal fibrosis of interlobular arteries.

**Table 5:** The association of morphologic variables of Oxford classification with scores of arteriolosclerosis and intimal fibrosis of interlobular arteries.

**N=136**	**Scores of arteriolosclerosis**	**Scores of intimal fibrosis of interlobular arteries**
**M**	P=N.S	P=N.S
**E**	P=0.16	P<0.001
**S**	P=0.028	P<0.001
**T**	P<0.001	P<0.001

**Table 6:** The association of deposited antibodies intensity score with scores of arteriolosclerosis and intimal fibrosis of interlobular arteries.

**N=136**	**Scores of arteriolosclerosis**	**Scores of intimal fibrosis of interlobular arteries**
IgA	P=N.S	P=N.S
IgG	P=N.S	P=N.S
IgM	P=N.S	P=N.S
C3	P=N.S	P=0.043

We did not correlate the other vascular lesions, such as TMA and small vessel vasculitis, with various clinical, laboratory and pathological data because the numbers of cases were very small.

## Discussion

The mechanism of IgAN progression is still ill-understood (1,7). Predictors for poor outcome comprise both clinical and histological findings. However, these factors may be better indicator of irreversible damage than the activity of the disease. The presence of kidney dysfunction, proteinuria and high blood pressure at the time of renal biopsy have been considered to be associated with the disease progression and correlated with interstitial fibrosis/tubular atrophy too (7-9). In a study on fifty-one patients with biopsy-proven IgAN, who were followed for 24 months, Brabcova *et al*. found that higher transforming growth factor-β1 (TGF-β1) and severe chronic vasculopathy were associated with the IgAN progression 24 months after biopsy. They suggested that, besides the known risk factors for chronic renal failure, advanced vasculopathy was associated with the IgAN progression (10). In our study,significant correlation between scores of arteriolosclerosis and intimal fibrosis of interlobular arteries with serum creatinine was observed. Also, the correlation of arteriolosclerosis with level of proteinuria was significantly positive. We also found, the significant positive association of arteriolosclerosis with S variant of Oxford classification and also a significant positive correlation of scores of interlobular arteries with scores of C3 deposition intensity. It is remarkable to notice that chronic vasculopathy, causes renal tissue ischemia and resultant, angiotensin II-dependent TGF-β1 overexpression, which was identified to be a critical component of renal fibrogenesis (10-12). TGF-β1 is a potent stimulus of the fibrosis leading to interstitial damage which is an important factor in severity of kidney injury and progressive renal failure (10-12). TMA occurs in IgAN, however, it is uncommon in the setting of this disease (13). The study published by ElKaroui *et al*. in Paris, showed that 53% of 128 IgAN patients had the morphologic lesions of TMA. They concluded that, lesions of TMA are frequent in IgAN and may occur in normotensive patients with near-normal renal histology (14). Our results are markedly different from the above findings. In our study, of 138 patients, only 2 had histopathologic features of TMA. Other investigators have also not reported this high prevalence. Therefore, further investigations are needed to determine whether IgAN has different his pathological features in different parts of the world. Furthermore, larger studies are also needed to determine the true frequency and clinical significance of other vascular lesions in patients with IgAN.

## Conclusion

The association of vasculopathy with serum creatinine and level of proteinuria support the role of vasculopathy in aggravation of IgAN. We also found a very low prevalence of TMA as compared with a French study, which needs more studies in larger series of IgAN.

## Author’s contributions

MM and HN wrote the manuscript equally.

## Conflict of interests

The authors declared no competing interests.

## Ethical considerations

Ethical issues (including plagiarism, misconduct, data fabrication, falsification, double publication or submission, redundancy) have been completely observed by the authors.

## Funding/Support

None.
